# Considerations for the risk of adverse health effects of different anatomy education modalities

**DOI:** 10.1007/s00276-022-03048-w

**Published:** 2022-11-19

**Authors:** Dimitrios Chytas, Maria Piagkou, Theano Demesticha, George Tsakotos, Konstantinos Natsis

**Affiliations:** 1 Basic Sciences Laboratory, Department of Physiotherapy, University of Peloponnese, Sparta, Greece; 2 Department of Anatomy, School of Medicine, National and Kapodistrian University of Athens, Athens, Greece; 3 Department of Anatomy and Surgical Anatomy, School of Medicine, Aristotle University of Thessaloniki, Thessaloniki, Greece

**Keywords:** Online anatomy education, Digital technology, Social media, Adverse health effect, Online anatomy teaching, Head-mounted displays

Dear Editor,

We read with great interest the letter by Mungmunpuntipantip and Wiwanitkit [4], who raised concerns about the use of online anatomy education. In this letter, it was highlighted that social media can cause adverse health effects (visual and hearing problems, or even seizures) to students. Respecting the authors’ opinion [4], we would like to point out that the paper discussed [1] by the authors did not focus on the use of social media in anatomy teaching. To the best of our knowledge, the literature lacks evidence that anatomy education, via social media, is significantly associated with adverse health effects to students. In contrast, we would like to highlight that the risk of students’ health problems seems to be associated with the mode of delivery of digital technology. There is evidence that the use of virtual or augmented reality (VR or AR) head-mounted displays (HMDs) in anatomy education has been accompanied by a higher risk of health complications, compared to the use of two-dimensional (2D) screens [2, 3]. In Moro et al. study [3], the participants, divided into three groups, were randomly allocated to receive anatomy teaching via a VR HMD (group 1), an AR 2D screen (group 2) and a tablet-based application (group 3). The examinations scores after the educational intervention, did not significantly differ. However, during the classes, the group 1 (VR HMD) was found significantly more likely to suffer from dizziness, headaches, and blurred vision, compared to the other groups which experienced 2D screen-based teaching. The participants of another study [2] were taught brain anatomy after having been randomly divided into two groups: the first one received teaching via an AR HMD and the second via an AR mobile handheld tablet device. Although the examinations scores were insignificantly different, the first group (AR HMD) experienced dizziness, in a significantly greater extent than the second.

Thus, we consider that the mode of delivery of digital technology in anatomy education does play a role in the risk of adverse health effects. Social media, or other digital anatomy teaching modalities, delivered via 2D screens, seem to carry a considerably lower risk for students’ health in comparison with HMDs. This fact does not mean that the educational benefit of HMDs should be ignored, but rather that anatomy teachers should use this technology with caution.

Further research is needed to clarify to what extent the use of digital technology may be safe. However, at present, it seems that social media, or other digital technologies delivered via 2D screens are less likely to cause adverse health effects than HMDs, when they are used for anatomy teaching purposes.

## Data Availability

Literature data are available if requested.
